# What Makes a Good Poster? Evaluating #BetterPoster and Classic Formats at a Scientific Cancer Conference

**DOI:** 10.1007/s13187-025-02622-1

**Published:** 2025-04-02

**Authors:** Line Bentsen, Daniella Elisabet Østergaard

**Affiliations:** 1 Department of Clinical Oncology, Copenhagen University Hospital - Rigshospitalet, Blegdamsvej 9, 2100 Copenhagen Ø, Copenhagen, Denmark; 2 Faculty of Health and Medical Sciences, Copenhagen University, Copenhagen, Denmark

**Keywords:** Scientific dissemination, Poster presentation, #BetterPosters, Education, Cancer research

## Abstract

Academic posters have evolved significantly, shifting from rare presentation opportunities to a common method of scientific dissemination. As speaking time and individualized attention for posters have diminished, new challenges have emerged, emphasizing the need for effective visual communication. This study aimed to evaluate the adoption and effectiveness of the #BetterPoster (BP) format compared to the classic format (C) and to determine whether the modified format contributed to an improved experience for both viewers and readers. At the national Danish Cancer Research Conference 2024, a total of 103 posters were categorized and assessed systematically by independent raters using four criteria: first impression, organization, poster format, and wordiness. Statistical analyses, including *t*-tests and Kendall’s *W* for interrater reliability, compared the formats, while Pearson correlations explored relationships between “first impression” and “organization,” “poster format,” or “wordiness.” Results indicated that the BP format achieved higher scores for all criteria, with significant differences in first impression (*p* = 1.64e-06). However, both formats exhibited potential for high scores when designed mindfully, balancing text and visual elements. While the BP format improved audience engagement, excessive simplification or disorganization reduced its effectiveness. Classic posters, although often more text-heavy, could also be effective when structured carefully. The findings highlight the importance of coherent design and training in visual communication, suggesting that well-executed posters, regardless of format, can enhance academic engagement and knowledge dissemination. These insights could inform future guidelines for academic poster presentations, fostering more engaging and effective communication at conferences.

**Supplementary Information:**

The online version contains supplementary material available at 10.1007/s13187-025-02622-1.

## Introduction

Academic communication is a complex and evolving discipline that requires disseminating specialized knowledge to diverse audiences. Among the many formats used to convey this information, the academic poster has undergone significant changes over time [1, 2]. Once considered a rare and prestigious opportunity to present at major conferences, posters have now become ubiquitous, with speaking time and individual attention for each poster significantly reduced. Despite their wide use, posters often occupy a lower status in the academic communication hierarchy and are frequently deprioritized.

This shift presents new challenges for the poster format, requiring it to balance multiple functions: creating an accessible overview, guiding the reader through the content, capturing attention among a variety of competing posters, and leaving an impression on the audience.

Recent literature has responded to these challenges by proposing new layout formats to enhance the effectiveness of posters as standalone communication tools. Specifically, the #BetterPoster approach offers an alternative to more classic formats, addressing the need for posters to be more visually engaging and reader friendly [3, 4].

In collaboration with the national Danish Cancer Research Conference (Danske Kræftforskningsdage) 2024, we have systematically explored how to improve the poster session, including refining guidelines for poster format inspired by the #BetterPoster framework. Based on this, we aim to assess the extent to which this new format has been adopted and whether it enhances the audience’s experience in academic settings.

The objectives of this study are twofold: [1] to evaluate the degree to which the modified format was applied and [2] to determine whether the modified format contributed to an improved experience for both viewers and readers, and what layout ideas were effectful.

### Method and Material

This is an observational study based on posters presented at the Danish Cancer Research Conference 2024, Odense, Denmark. The conference is multidisciplinary but solely focused on cancer research, approached with multiple perspectives and methodologies. The conference attendees included researchers, healthcare management, policymakers, journalists, patients, advocacy groups, and health authorities (total number of guests 534). A total of 103 posters were accepted for the conference. Poster presenters prior to the conference were strongly encouraged to follow the official guidelines for poster format, which was inspired by #BetterPoster format by Morrison et al. and was sent out as a poster guideline to the presenters (available in supplements) [3, 5]. Posters were visible for the full length of the conference (two full days). At the end of the first day of the conference, an interactive poster session was schedule with a duration of 1 h. Here, posters were orally presented on ten simultaneously poster tracks, each track moderated by two moderators to facilitate questions and discussion.

#### Categorization of Poster Format

All posters were categorized based on their format as either “#BetterPoster format” in coherence with Morrisson’s format ideas (bill board conclusion/take-home message, important figures or messages centralized, format guiding the reader through the study) [3, 4] or as “classic format” following a traditional format (title at the top, IMRAD (introduction, method, results, and discussion), figures, and conclusion at the bottom of the paper) [6]. The categorization was done independently by two raters (author LB and DEØ), and if there were disagreements, the poster was reassessed together, and consensus was reached.

#### Items Assessed

All posters were assessed for four items: first impression (0–50 points), organization (1–4 points), poster design (1–4 points), and wordiness (1–4 points).

The four items were assessed by two to four raters per poster. Authors LB and DEØ rated all posters independently, and the moderators who moderated the poster presentations at the interactive poster session were invited to rate the posters in their track independently.

For the item “first impression,” an arbitrary scale of 0–50 points was used where 50 was the best score. It was based on the visual first impression of the poster [7, 8].

A detailed description of the scales for the items “organization,” “poster design,” and “wordiness” were defined as seen in Table 1, inspired by Khadka et al. [8]. All raters had received written information about the items and scales before the conference.

**Table 1:** Rating of items

Item	Score			
	**Exemplary (4 points)**	**Acceptable (3 points)**	**Sub-par (2 points)**	**Poor (1 point)**
**Organization**	Information clean straightforward, organized	Some left to be desired/better	Much left to be desired/better	Neither clean nor straightforward
**Poster design and use of graphics**	Visually helpful, eye catching, pleasant to eyes	Some left to be desired/better	Much left to be desired/better	Visually unpleasant
**Wordy or busy**	Not busy or wordy (easy to review/understand)	Slightly busy or wordy (some wordiness present but can be easily reviewed/understood)	Busy and/or wordy (majority was text, difficult to review quickly)	Very busy and/or wordy (full of text, some vague, some ambiguous)

Adapted from Khadka et al. (2024)

#### Statistical Analysis

Data analysis was performed using R (version 4.3.2). Poster scores were collected from up to four raters per poster across the four items, and variations depending on the number of raters were assessed before the statistical analysis. Interrater reliability was assessed using the non-parametric test Kendall’s *W* for all four item scores across all four raters [9]. The Shapiro–Wilk test and Levene’s test were used to assess the normality and homogeneity of variances for all four items, respectively. *t*-tests were performed to compare scores for all four items for the two poster types, and a difference was considered significant if the *p*-values < 0.05. If assumptions for parametric testing were not met, the non-parametric Wilcox test was used instead. Pearson correlations were calculated to explore the relation between “first impression “ and “organization,” “poster design,” or “wordiness.”

## Results

A total of 103 posters were included in this study. Each poster had a minimum of 15 visitors during the interactive poster session; however, most had > 25 visitors.

Variance of mean scores on each item based on number of raters was evaluated with the standard deviation and Levene’s test which showed no significant difference for two, three, or four raters for all four items. Plots are available in supplementary.

### Interrater Reliability

Overall, the test for interrater reliability showed moderate to strong agreement for three items (first impression, poster design, and wordiness) and low agreement for one item (organization). Items with strong and moderate agreement were significant in agreement whereas the test for organization was not significant (table S2 for values).

### Poster Type and Scores

The initial consensus for categorizing the posters format was 57%. Consensus for all posters was reached with a second joint assessment (by author LB and DEØ).

**Fig. 1:**
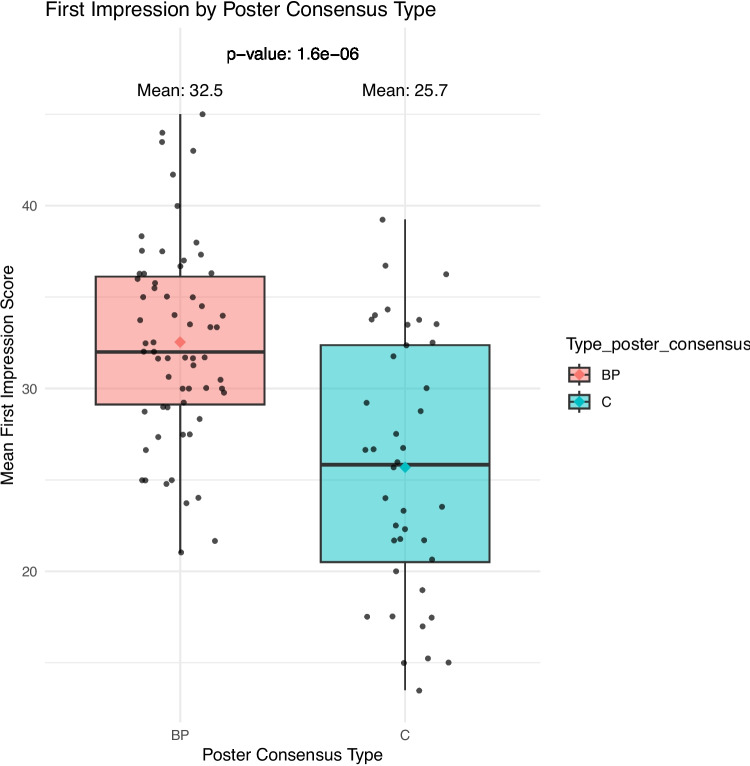
Boxplot of the scores for first impression (scale 0–50 point) divided in the two formats—better poster or classic format. *T*-test performed, *p*-value = 1.64e-06, meaning #BetterPoster format significantly had higher scores for first impression

The mean scores for “first impression” for #BetterPoster and classic format were 32.5 and 25.7, respectively. A *t*-test showed significantly higher scores for #BetterPoster format (*p*-value = 1.64e-06). The individual scores for first impression for both poster formats are visualized with a jitter-box plot, cf. Figure 1.

**Fig. 2:**
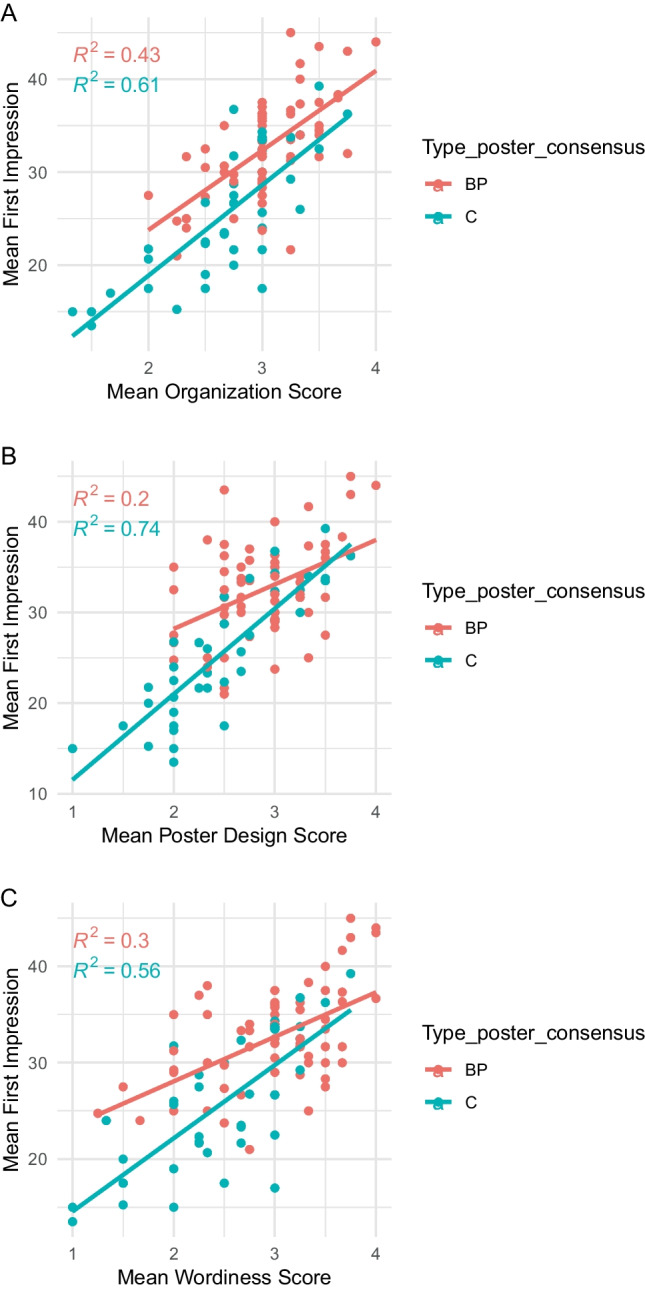
Three correlation plots of assessed items (organization, design, and wordiness) vs. first impression. **A** First impression score correlated to scores for item “organization” correlated to. **B** First impression score correlated to scores for item “poster design” and **C** first impression score correlated to scores for item “wordiness.” Overall, the plot shows that the higher the score for organization, design, or wordiness, the higher the score for first impression

Pearson’s correlation was then applied to assess how the first impression was related to organization, poster design, and wordiness. This is visualized in Fig. 2A–C. The correlation plots show that in general, posters using better poster format had higher scores compared to poster using classic format. However, the linear regression model was steeper for classic compared to better poster format. Statistical testing for difference in scores for the two poster formats for all four items showed significantly higher scores for the better poster format (table S3).

## Discussion

In this study, based on the poster session at the national Danish Cancer Research Conference 2024, we found that the better poster format vs. the classic format scored significantly higher for first impression, organization, poster design, and wordiness. Interrater reliability was strong to moderate level for three items and low for the item “organization.” Linear regression indicated that the mindful use of any poster format would result in higher first impression scores for both better poster and classic formats.

While the better poster format aims to simplify content with minimal text and more visual elements, we discovered that lower rating also appeared among posters in this format. Firstly, reducing text too much did not always translate into clarity. Posters with better poster format could become disorganized, especially if random elements like excessive highlighting or ill-fitting images were used. In contrast, the classic format faced the opposite problem—lower rating was often associated with the poster being overly text-heavy. Many posters with classic format featured long blocks of text with conclusions placed at the bottom, requiring viewers to sift through a great deal of information to grasp the key message. Secondly, many posters used boxes to frame sections, and while this is a step towards guiding the audience, too many boxes will eliminate the guidance, just as a text heavy box will make readers skip or skim this part, and boxes with no text will leave the audience puzzled. Our results indicated that both poster formats could reach high scores for all items, which indicated that mindful poster layout is essential for clear and engaging scientific dissemination.

Our findings align with several studies examining the visual impact and effectiveness of posters at academic conferences [10–12]. Similar to our results, Khadka et al. (2024) found that while contemporary poster formats, such as the better poster format, appeared more helpful in engaging the audience, they were not without flaws. Their study emphasized that the quality of posters varied significantly, ranging from poorly executed to exemplary, regardless of format [8].

Other researchers have emphasized the importance of first impressions at academic conferences. When attendees are presented with a vast number of posters, the initial visual impression often determines whether they engage with the content or move on [7, 13]. Another study showed that only a small proportion of conference attendees take the time to read posters in detail or ask questions to the presenters [14]. This reinforces our finding that posters with a strong first impression, such as those mindfully using the better poster or classic format, are more likely to capture attention and facilitate further engagement.

Our results also support the argument that poorly organized posters, regardless of poster format, can appear confusing and difficult to understand as it increases the cognitive load [15]. The poster format is one of many formats used for scientific dissemination at conferences. Previous research advocates for the use of structured formats, such as the IMRAD format (introduction, methods, results, and discussion), which improves clarity and aids in the logical flow of information and ensures proper conduct of research [6]. However, limiting the focus in the poster to two or three key findings is crucial for an effective research poster [16].

**Fig. 3:**
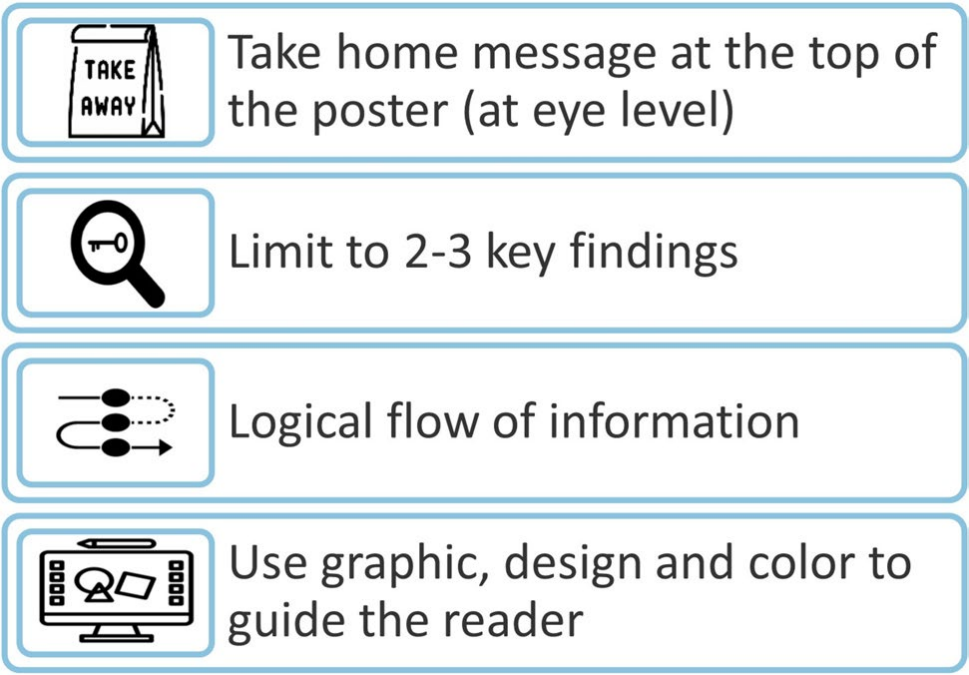
Highlights: The content of the poster must be organized to guide the reader and translate the results into real world/clinical settings

Therefore, based on the results from this study, we argue that both formats have their merits, and a well-executed poster borrows elements from both. A poster layout is most successful when it adheres to a coherent guidance of the reader. This includes the key “take-home” message displayed at the top of the poster to ensure that the most important information is communicated immediately. Additionally, ensure a clear structure, use bullet points to summarize sections succinctly, and include a few carefully chosen illustrations to enhance understanding without overwhelming the audience. These points are summarized in Fig. 3. In addition, a notable issue in poster preparation is the lack of formal training in communication and visualization of research with respect to different types of communication styles (scientific paper, news article, web pages, posters, etc.) [17–19]. Jambor et al. found that most researchers receive no design training and often receive little to no feedback when preparing their visual materials for poster presentations [20]. This lack of guidance leaves many researchers struggling to effectively visualize their findings. Addressing this gap in training could lead to more compelling and visually coherent posters, which are critical for engaging an academic audience as well as collaborators from relevant industries.

### Strengths and Limitations

There are limitations to our study that should be noted. Firstly, the subjective nature of the evaluations, especially with certain moderators (including LB and DEØ) having a preference of the better poster format, may have influenced the scores and remains as a potential source of bias. Furthermore, we did not conduct repeated evaluations of the posters after a period of time, which could have provided insights into the consistency of our assessments. The traffic for each poster during the whole conference was not measured systematically. Finally, LB and DEØ have previously encouraged poster presenters to adopt the better poster format, which may have contributed to some degree of bias, although this influence did not extend to the other raters. While additional forums of varying sizes would increase the generalizability of our findings, our study offers a pragmatic and feasible approach to assess a recent paradigm shift in poster formats, contributing to evidence-based guidance for scientific dissemination.

## Conclusion

In conclusion, while the better poster format generally yielded higher scores for first impressions and overall visual appeal, both poster formats can be effective if properly executed. The key lies in balancing text, visuals, and structure to communicate the research in a clear, guiding and engaging way.

## Supplementary Information

Below is the link to the electronic supplementary material.

ESM 1 (1. 21 MB PDF)
