# Supplementary material for: What Makes a Good Poster? Evaluating #BetterPoster and Classic Formats at a Scientific Cancer Conference
Source: J Cancer Educ. 2025 Apr 2;41(1):49–54. doi: 10.1007/s13187-025-02622-1 (PMC12971741; doi:10.1007/s13187-025-02622-1)
Supplement: Supplementary file 1 — (1. 21 MB PDF) [file 13187_2025_2622_MOESM1_ESM.pdf]

Supplementary:

Guideline for poster format used at Danske Kræftforskningsdage 2024

Attached as pdf file.

Variance of mean scores by number of raters

Tabel S1.: Levene test number of raters	p-value
Mean First impression	0.3814
Mean Organization	0.1965
Mean Poster design	0.2006
Mean Wordiness	0.6033

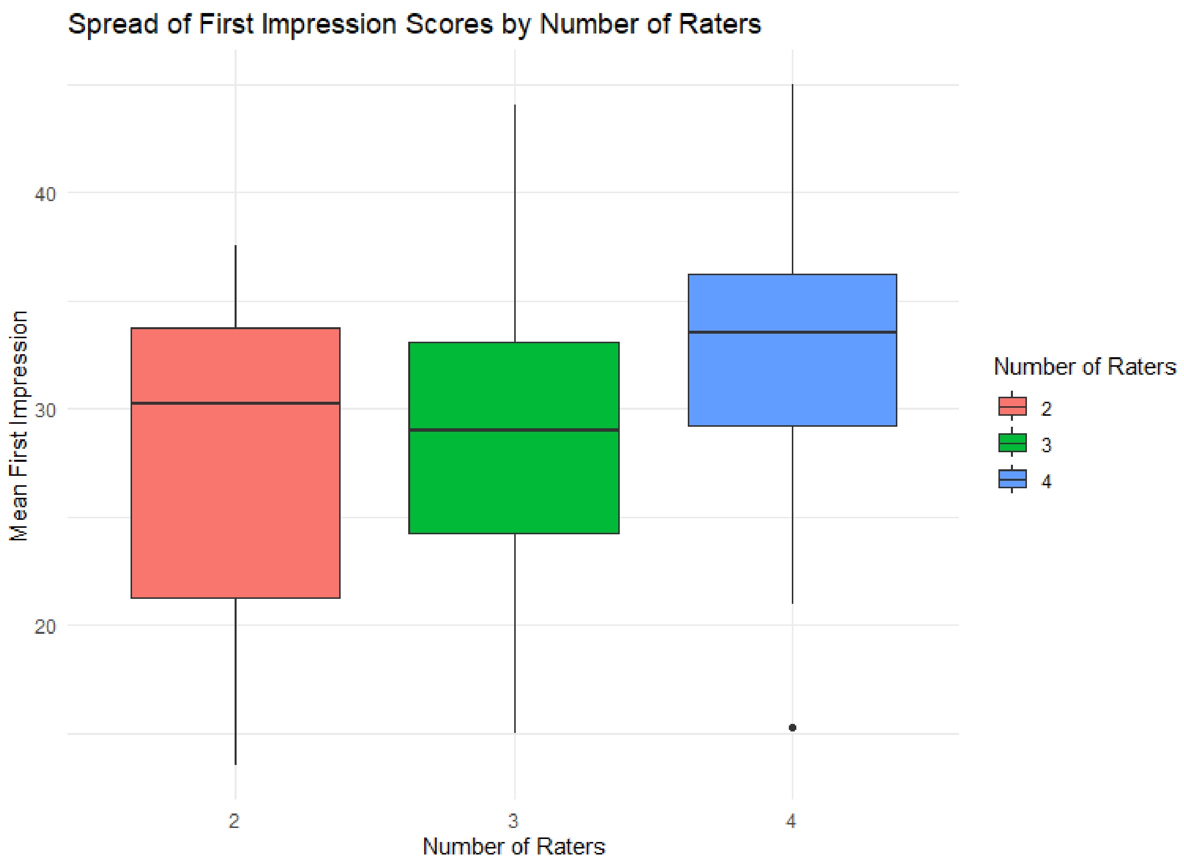

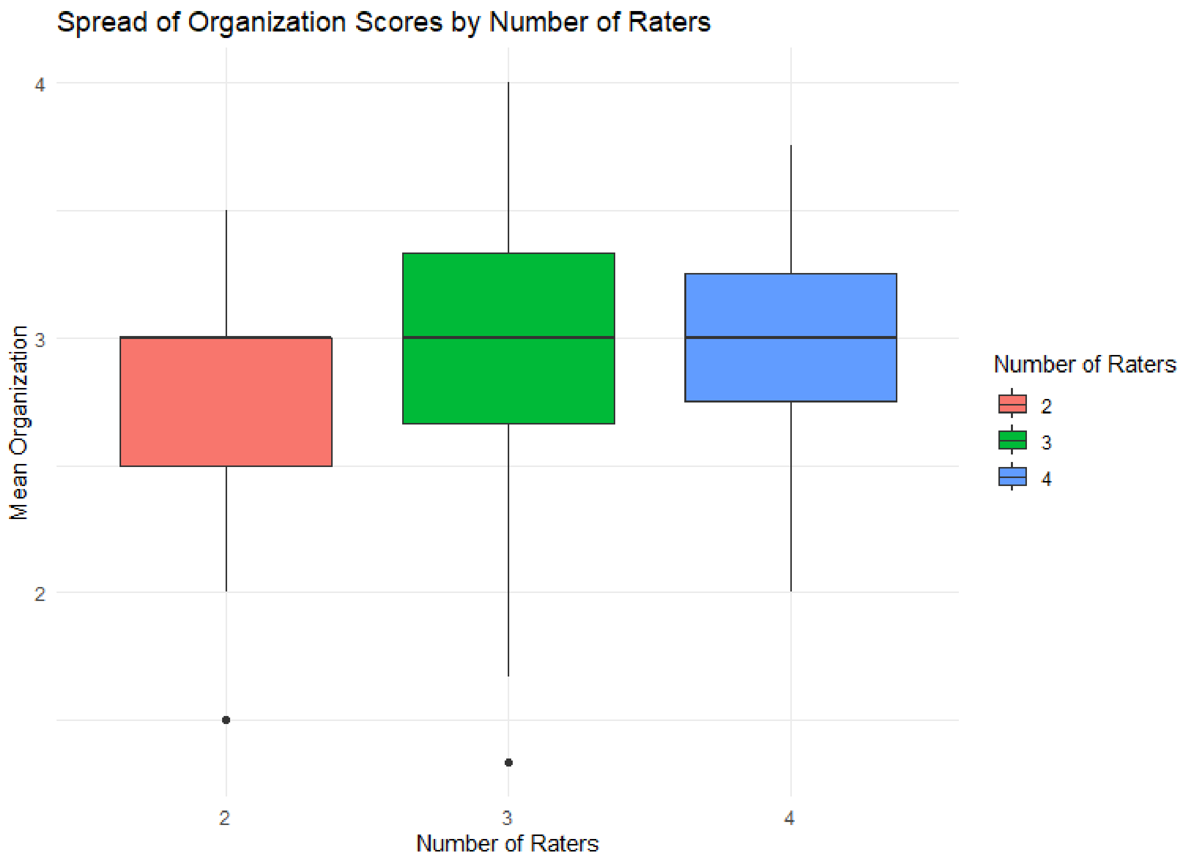
Spread of Organization Scores by Number of Raters
Mean Organization
4
3
2
2
3
4
Number of Raters
Number of Raters
2
3
4

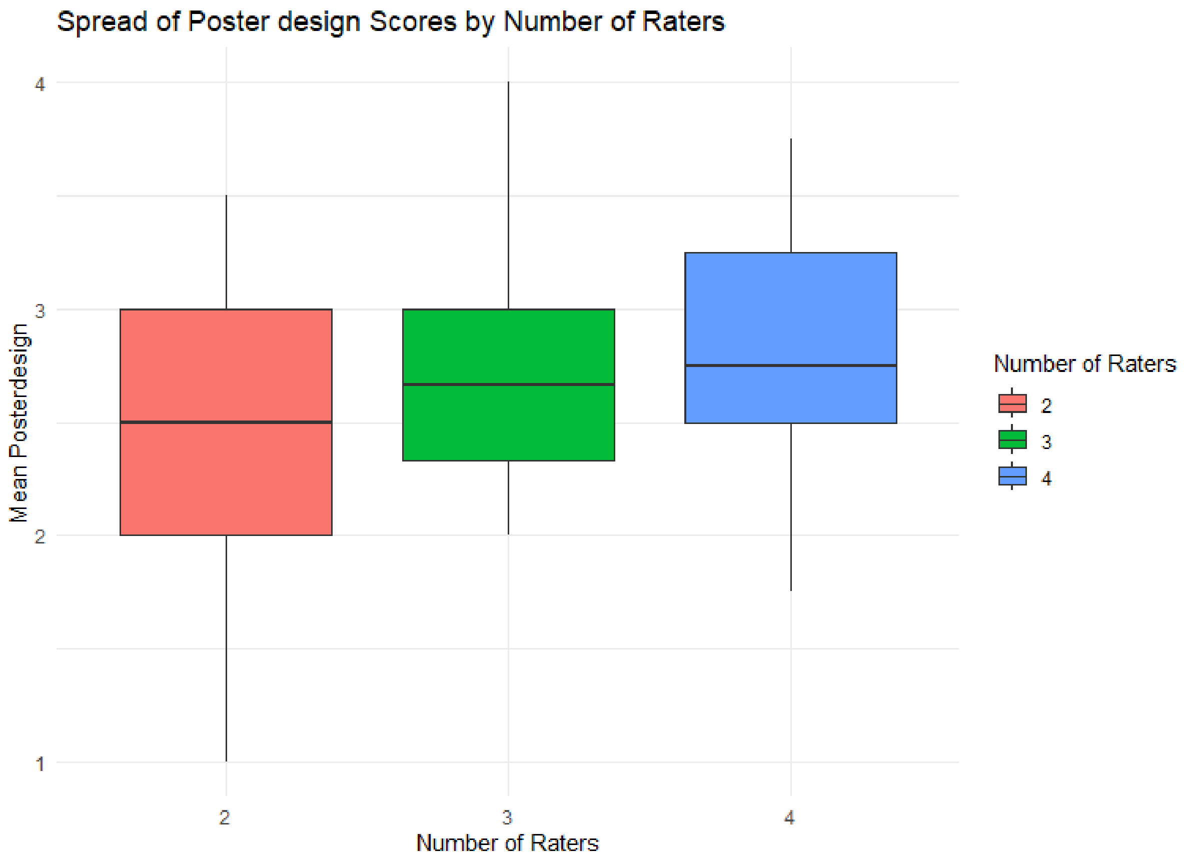
Spread of Poster design Scores by Number of Raters
Mean Posterdesign
4
3
2
1
2
3
4
Number of Raters
Number of Raters
2
3
4

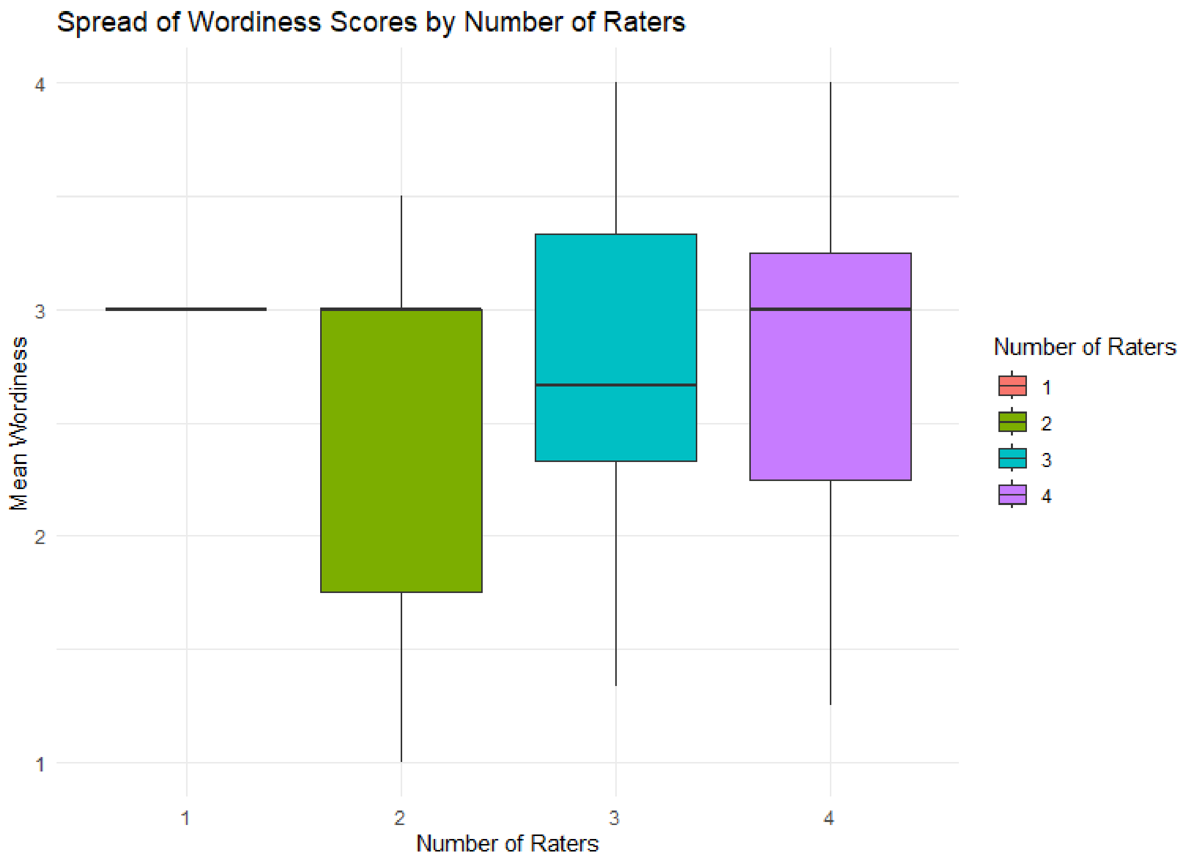

Interrater reliability

Table S2<br>Item	W	Ties	p-value	
First impression	0.43		0.00422	Moderate level of agreement, agreement is significant
Organization	0.243		0.531	low level of agreement, not significant
Poster design	0.377		0.0109	moderate level of agreement, agreement is significant

Wordiness	0.468		0.000159	strong level of agreement, agreement is significant

Table S3: Item	Mean value		p value	95% CI
	BP	C		
First impression	32,5	25,7	1,64 e-6	4,2485 - 9,417
Organization	3,0	2,7	0,0026 (t-test)<br>0,003* (wilcox test)	0,1178 - 0,529
Poster design	2,9	2,5	0,0015	0,1547 - 0,6272
Wordiness	3,0	2,5	0,0004 (t-test)<br>0,0003 (wilcox test)	0,2333 - 0,7740

Put Your Full Poster Title Here: *And Include Your Subtitle if You Have One*

Put your logo/affiliate/ hospital/university /etc- here →

Main finding goes here, translated into **plain English.**

Emphasize the important words.

(feel free to add a relevant graphic below!)

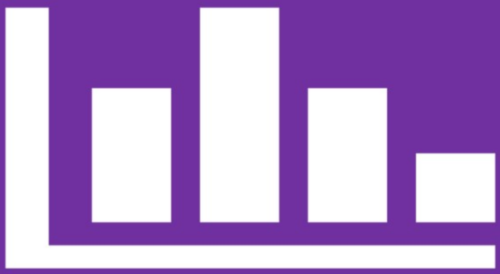

email@address.dk
If full paper available put in link or QR code

Author Name1, author2, author3, author4

INTRO

- Explain why your study matters in the fastest, most accessible way possible to the audience (feel free to add graphics (with alternative text)!).

METHODS

1. How did you find this?
2. Collected [what] from [population] etc.
3. How you tested it.

RESULTS

- Graph/table/text with **essential results only**
- All the other data in ”SUPPORTIVE DATA/PLOTS/ETC”

DISCUSSION

- What did you find? Is it new? Is it true? Put your results/findings into context but be concise and minimal in text.
- Bullet points can be a nice tool here.

SUPPORTIVE DATA/PLOTS/ECT

Delete this and replace it with your…

- Extra Graphs
- Extra Tables
- Extra Figures
- Extra nuance that you’re worried about leaving out

This section is for the data that will support your findings for the data interested audience.

This is our version of APA mod and Mike Morrison’s Better Poster. Keep what works for you. Change what doesn’t.

Tips by Mike Morrison:

1. Keep **font size** as high above **28+** as possible.
2. Keep your summary tight. **Think** of it like “**abstract+**” with key figures only.
3. The **more content you add here**, the **more cognitive load** you add, and the more you’ll turn people off engaging.
4. *Less* content = *more* readers.
5. Now **delete this** text box. ☺
